# Orthopedic Manifestations of Bruck Syndrome: A Case Series with Intermediate to Long-term Follow-Up

**DOI:** 10.1155/2019/8014038

**Published:** 2019-03-13

**Authors:** Adolfredo Santana, Geovanny Oleas-Santillán, Jeanne M. Franzone, L. Reid Nichols, J. Richard Bowen, Richard W. Kruse

**Affiliations:** Department of Orthopedic Surgery, Alfred I. duPont Hospital for Children, Wilmington, DE, USA

## Abstract

The aim of this study was to evaluate the association of contractures, fractures, and deformities in four patients with Bruck syndrome treated in our facility. Data were collected from medical records, radiographs, dual-energy X-ray absorptiometry (DEXA) scans, genetic tests, and gait analysis. All had contractures at birth and genotypic findings including mutations in PLOD2 or FPKB10. Three cases were treated with bisphosphonates with improvement in bone density verified by DEXA. In Bruck syndrome, orthopedic deformities include the following sequential aspects: contractures, characterized by upper and lower extremity contractures such as clubfeet; fractures, characterized by multiple diaphyseal fractures in the long bones of the extremities; and deformities, characterized by malalignment of extremities and the spine. Physical therapy and bracing proved helpful for the contractures to try to stop progression. Bone fragility needs to be considered when deciding to attempt cast correction. Surgeries in the soft tissues can be performed to retain joint movement. In fractures with angulation, intramedullary nail fixation was useful, and in cases without deformity, casting alone was successful. We suggest monitoring the bone density with DEXA, nutrition support with vitamin D and calcium, and treatment with bisphosphonates. Spine deformities were successfully treated by spinal fusion and instrumentation.

## 1. Introduction

In 1897, Alfred Bruck described a syndrome, which was characterized by congenital joint contractures and bone fragility [1, 2]. Bruck syndrome has been regarded as an autosomal recessive form of osteogenesis imperfecta (OI) and is associated with collagen folding and cross-linking defects in the FKBP10 and/or PLOD2 genes [3–6]. The phenotype is indistinguishable between the two genotypes. Breslau-Siderius et al. suggested that Bruck syndrome is not a subtype of OI or arthrogryposis multiplex congenital (AMC) but rather is a distinct disorder [7].

The rarity of this syndrome results in an incomplete description of both the disease characteristics during growth as well as the outcomes of treatment. The aim of this study is to evaluate the association of contractures, fractures, deformities, and various treatments of four patients with Bruck syndrome.

## 2. Case Presentations

After the Institutional Review Board approval, a retrospective review of patients identified with Bruck syndrome treated at one facility from 2002 to 2017 who had a PLOD2 and FPK10 mutation was undertaken. Demographics, clinical data, radiographs, dual-energy X-ray absorptiometry (DEXA) scans, and gait analysis were collected. Data were organized in a time-related sequence to understand the orthopedic aspects of this syndrome.

### 2.1. Case 1

This is a 7-year-old male patient, with a diagnosis of Bruck syndrome proven by genetic testing (gene FKBP10 mutations: c.449G>A, p.Trp150Ter). He has no family history of OI or AMC, parental consanguinity, dentinogenesis imperfecta, blue sclera, or hearing loss and a birth weight of 3.2 kg. He was treated with bisphosphonates (pamidronate, 1 mg/kg/dose intravenously for three days per cycle with a cycle every four months) for five years and his most recent DEXA scan showed a lumbar z-score of −7, 4. Currently, he is nonambulatory and uses a power wheelchair. He has flexion contractures of both hips with a range of motion to 45 degrees on the right and 35 degrees on the left. Knee contractures limited the range of motion to 60 degrees on the right and 70 degrees on the left. His right ankle was dorsiflexed to neutral, and his left ankle to 30 degrees of dorsiflexion and 30 degrees of plantarflexion. Finally, his right foot had -10 degrees of abduction and 10 to 40 degrees of adduction, and his left foot had -10 degrees of abduction and 10 to 50 degrees of adduction.

**Figure 1:**
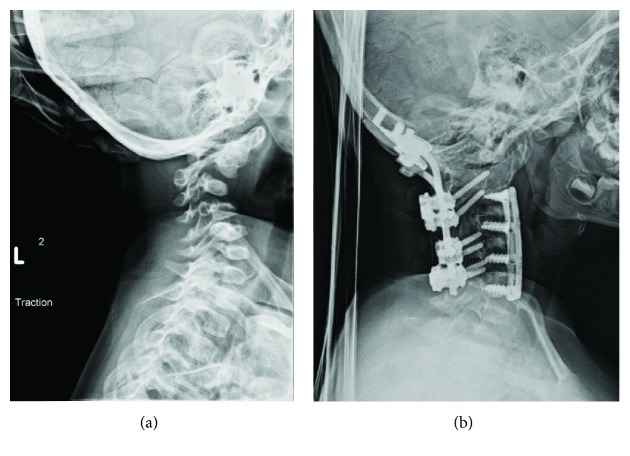
(a, b) Case 1. (a) Cervical spine kyphosis, no wormian bone in the skull, and (b) spinal fusion from occiput to C5.

He developed multiple orthopedic problems. His cervical spine developed progressive kyphosis with no basilar invagination. He required a spinal fusion from occiput to C4 at the age of 4 years, and recurrence of the deformity required revision spinal fusion from C1 to C5 at the age of 5 years (Figure 1). Kyphoscoliosis and thoracic deformity (“barrel chest” deformity) developed and progressed during growth to 45 degrees by the age of 7 years.

Flexion contractures of the upper limbs were present at birth with limited elbow range of motion to 5 degrees on the right and 15 degrees on the left, wrists bilaterally, and left fifth finger. At the age of 7 years, he had a right ulna fracture that was treated by casting.

In his lower limbs, he had flexion contractures of the hips and knees at birth and bilateral clubfoot. Subsequently, he had multiple fractures in his lower extremities that were treated by operative intramedullary stabilization and had subsequent recurrence of deformities with growth requiring revisions. At the age of 2 years, he had intramedullary Rush rod fixation in the left femur and tibia. At the age of 3 years, the left femur recurred and he underwent revision with new intramedullary Rush rod fixation. At the age of 5 years, he had intramedullary stabilization of the right femur and revision of the left femur as well as left tibia intramedullary Rush rod fixation. At the age of 6 years, he underwent right tibia intramedullary Rush rod fixation and revision of the right femur with dual Rush rods. At the age of 7 years, he underwent left femoral and tibial rod exchange with Fassier-Duval rods (tibia with dual-locking plates and bone grafting). He had bilateral clubfoot treated with serial casting using the Ponseti method as an infant with deformity that relapsed until at the age of 7 years when he underwent bilateral Achilles tenotomies (Table 1).

### 2.2. Case 2

This is a 24-year-old female patient with Bruck syndrome proven by genetic testing (defects in PLOD2, mutation: c.517G>C; p. Alal73Pro). She has no family history of OI or AMC, parental consanguinity, dentinogenesis imperfect, blue sclera, or hearing loss and a birth weight of 2.5 kg. However, she has micrognathia. She ambulates with bilateral ankle-foot orthotics. She received bisphosphonate (pamidronate, 1 mg/kg/dose intravenously for three days per cycle with a cycle every four months) therapy for five years. Her last DEXA scan was normal for her age.

**Figure 2:**
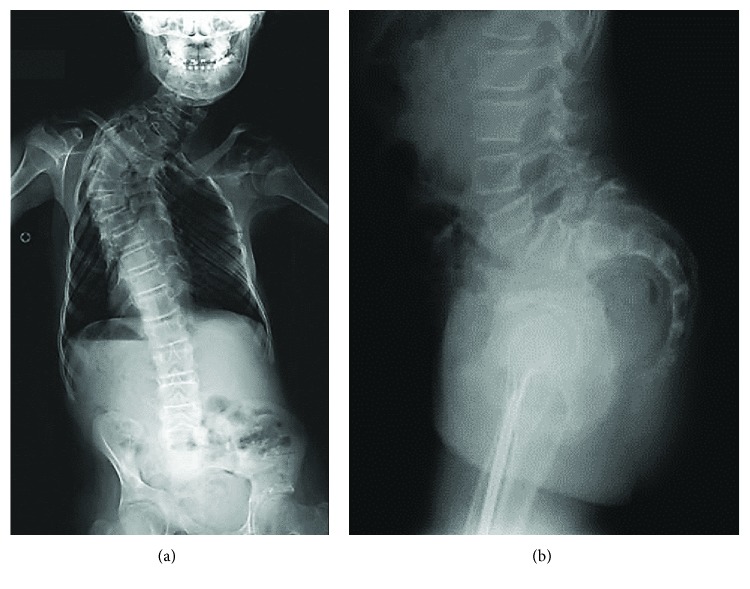
(a, b) Case 2. 12 years old with (a) scoliosis with a thoracic and lumbar and (b) spondylolisthesis at L5 and lumbar hyperlordosis.

Joint contractures were present in the upper and lower limbs from birth. The range of motion of both shoulders was limited to 90 degrees of flexion. Her hips were in 90 degrees of flexion with limited hip abduction, and her knees were in -30 degrees of extension. At 12 years of age, coliosis developed in the spine with a thoracic curve of 51 degrees and lumbar curve of 27 degrees. She also had spondylolisthesis at L5 and lumbar hyperlordosis (Figure 2). At the age of 13 years, she had a posterior spine fusion from C7 to T10.

In her upper limbs, she fractured her right olecranon at the age of 14 years. This fracture was treated by casting. At the age of 16 years, she fractured her right proximal humeral shaft, which was treated by an intramedullary flexible nail. Subsequently, at the age of 23 years, she developed left ulna nerve compression (cubital tunnel syndrome), which was treated by operative decompression and subcutaneous transposition.

**Figure 3:**
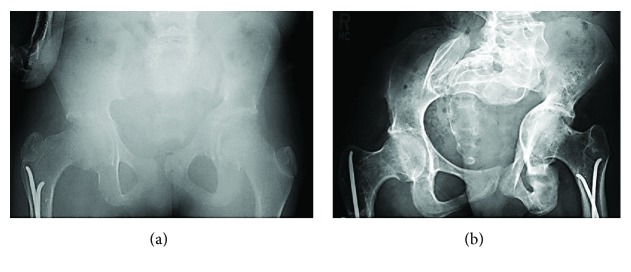
(a, b) Case 2. 14 years old with development of left acetabular protrusion.

In the lower limbs, she had bilateral clubfoot treated by casting. Operative realignment was performed on her right foot at the age of 2 years. At the age of 9 years, she fractured her right femur, which was treated by two intramedullary flexible nails. At the age of 11 years, she refractured the diaphysis of the right femur and bent the intramedullary rod, which required revision with repeat intramedullary flexible nailing. Her left femur was fractured when she was at the age of 12 years; this was treated with two flexible nails. At the age of 14 years, she developed left acetabular protrusio (Figure 3). At the age of 21 years, her right femur was fractured in the diaphysis, which was treated by a locking plate-intramedullary rod construct. The plate was removed when she was at the age of 22 years (Table 1).

### 2.3. Case 3

This is a 6-year-old male patient, with a suspected diagnosis of Bruck syndrome at the second day of life, proven by genetic testing (defects in PLOD2 and FKBP10, mutation: c.831dupC). There is no family history of OI or AMC, dentinogenesis imperfecta, blue sclera, parental consanguinity, or hearing loss and a birth weight of 2.8 kg. This child is also nonambulatory and uses a wheelchair in the community. He has hip flexion contractures of 15 degrees on the right and 25 degrees on the left). He has knee flexion contractures with popliteal angles of 30 degrees on the right and 40 degrees on the left. He has bilateral elbow flexion contractures of 10 degrees. His right foot is internally rotated, and his left foot is externally rotated. He uses bilateral knee-ankle-foot orthotics for support. He was treated with bisphosphonate (pamidronate, unknown dosage) from the age of 6 months to 3 years at another hospital. His last DEXA scan was normal for this age.

**Figure 4:**
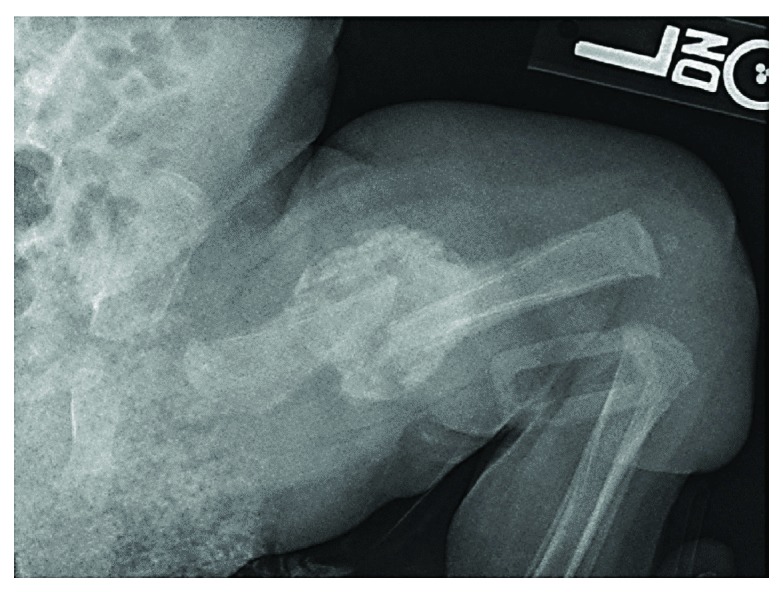
Case 3. 6 weeks of age with left femur fracture healed with voluminous callus.

**Figure 5:**
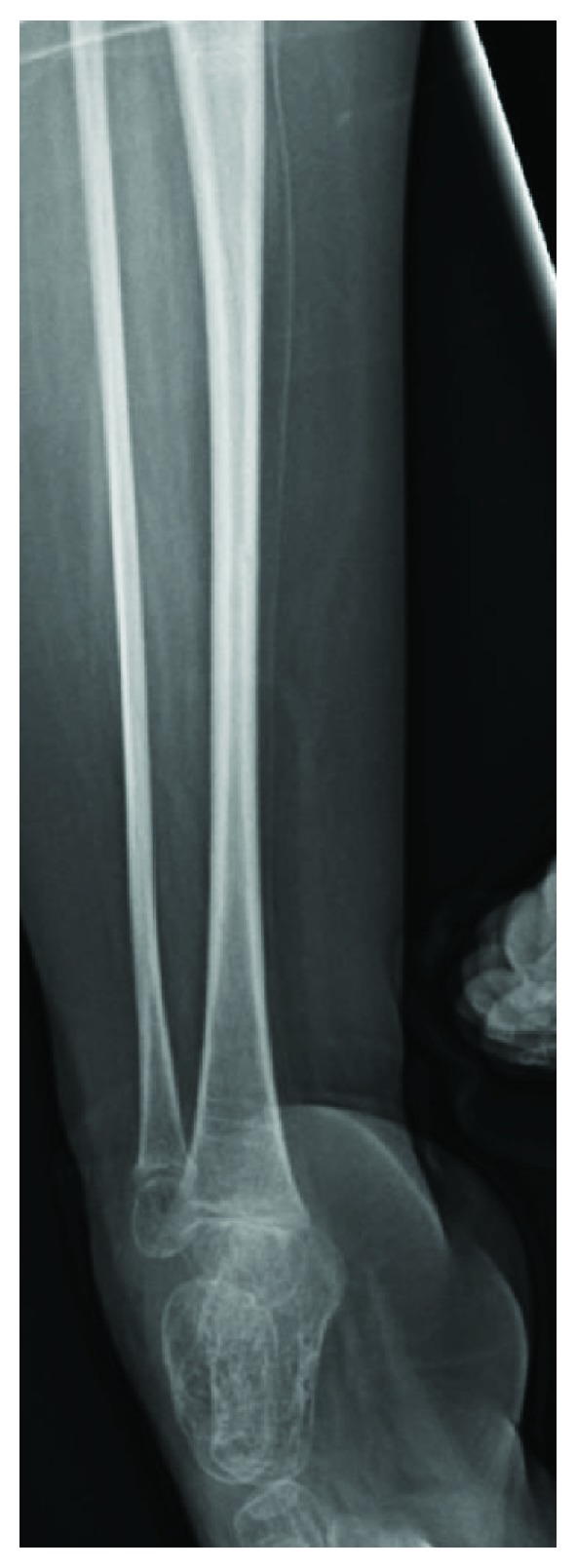
Gracile shape of the right tibia showing the bones becoming narrower in the diaphysis.

At 6 weeks of age, he developed fractures in the vertebral bodies and six right ribs, right clavicle, right radius, and bilateral femurs. The fractures healed with voluminous callus (Figure 4). Subsequently, the callus remodeled normally, and the limb bones grew into a gracile shape, appearing narrow at the cortex (Figure 5). At the age of 3 years, he had a right midshaft femoral fracture treated with spica casting. He had a left midshaft femoral fracture at the age of 4 years treated with splinting (Table 1).

### 2.4. Case 4

This is a 4-year-old male patient with a diagnosis of Bruck syndrome at birth. There is no family history of OI or AMC, parental consanguinity, dentinogenesis imperfecta, blue sclera, or hearing loss and a birth weight of 2.9 kg. Currently, he is nonambulatory and uses a wheelchair for all mobility activities. In his upper limbs, he had flexion contractures at birth of the elbow and wrist. The left upper limb is the most involved with contractures. His left elbow is unable to be extended past 90 degrees and the wrist beyond 30 degrees. In his lower limbs, he had a right midshaft femur fracture at birth and midshaft nondisplaced right tibia fracture at the age of 4 years. Both fractures were casted and no orthopedic surgeries have been required (Table 1). There was no treatment with bisphosphonates.

## 3. Discussion

In our study, we have presented a pattern of disease during growth that includes three aspects of orthopedic considerations: contractures from birth, fractures during growth, and subsequent deformities. All children had contractures of both the upper and lower limbs at birth. We recommend physical therapy for contractures and serial casting for clubfeet; however, bone fragility needs to be considered when applying casts with manipulation. Soft tissue surgeries may help to retain joint movement.

Fractures typically develop in the diaphysis of both the upper and lower limbs. Fractures healed with adequate callus formation, and the rate of healing appears to be normal. We suggest monitoring the bone density with DEXA, nutritional support with vitamin D and calcium, and treatment with bisphosphonates [3, 8–14].

Deformities in the upper and lower limbs develop from recurrent fractures. The deformities in the limbs were treated successfully by osteotomies and intramedullary stabilization. Spine deformities such as scoliosis, kyphosis, and spondylolistheses developed during growth and when severe, were treated by spinal fusion and instrumentation [15, 16].

In summary, the orthopedic manifestations of Bruck syndrome consist of the following sequential aspects: contractures (characterized from birth in the upper and lower extremities to include clubfeet), fractures (characterized by multiple diaphyseal fractures in the long bones of the limbs), and deformities (characterized by malalignment of extremities and the spine). Contractures from birth may result in an inappropriate diagnosis of arthrogryposis. However, changes with growth, bone fragility resulting in fractures, and deformities in the spine and limbs confirm the diagnosis. Treatment by physical therapy, orthosis, and orthopedic procedures is helpful. Also, we suggest monitoring the bone density with DEXA, nutritional support with vitamin D and calcium, and treatment with bisphosphonates.

## Figures and Tables

**Table 1:** Orthopedic aspects in Bruck syndrome.

	Contractures		Fractures		Deformities	
	0-28/30 days	1-12 months	1-3 years	4-6 years	7-12 years	13-18 years
*Case 1*						
Contractures UL	Elbows, wrists, fifth digit	Elbows, wrists, fifth digit	Elbows, wrists, fifth digit	Elbows, wrists, fifth digit	Elbows, wrists, fifth digit	Elbows, wrists, fifth digit
Contractures LL	Clubfeet, hips, knees bilateral	Hips, knees	Hips, knees	Hips, knees	Hips, knees	Hips, knees
Spine deformity	None	None	None	Cervical kyphosis	Thoracolumbar kyphosis	None
UL fracture/deformity	None	None	None	None	Right ulna fracture	None
LL fracture/deformity	None	None	Left femur and tibia (Rush rod)	Bilateral femur (dual Rush rods)	Left femur and tibia (Fassier-Duval)	None
*Case 2*						
Contractures UL	Shoulders	No changes	No changes	No changes	No changes	No changes
Contractures LL	Clubfeet bilateral hips and knees	Hips, knees	No change	No changes	No change	No changes
Spine deformity	None	None	None	Mild scoliosis	Scoliosis, spondylolisthesis L5, lumbar hyperlordosis	Posterior fusion
UL fracture/deformity	None	None	None	None	None	Right olecranon fracture Right humeral shaft fracture (intramedullary nail)
LL fracture/deformity	None	None	None	None	Right femur fracture (flexible nails) Refractured—bent rod (renail) Refracture (locking plate-intramedullary rod) Left femur fractured (flexible nails)	none
*Case 3*						
UL contracture	None	None	None	None	None	None
LL	Bilateral knee	Bilateral knee flexion contractures 50°	Bilateral knee	Hips, knees	Hips, knees	Hips, knees
Spine deformity	None	Vertebral, rib fractures	None	None	None	None
UL fracture/deformity	None	Clavicle, radius	None	None	None	None
LL fracture/deformity	None	Bilateral femur fractures	Right femoral fracture	Left femoral fracture, foot rotated	None	None
*Case 4*						
Contractures UL	Left upper	Left upper	No change	No changes	No change	No changes
Contractures LL	Hips and knees	No change	No change	No changes	No change	No changes
Fractures and deformities						
Spine deformity	None	None	None	None	None	None
UL fracture/deformity	None	None	None	None	None	None
LL	Right femur fracture (cast)	None	None	Right tibia fracture (cast)	None	None

LCP, locking compression plate; LL, lower limb; OC, occiput; UL, upper limb.
